# Mindfulness-based stress reduction as perceived by individuals with pathological mental fatigue after an acquired brain injury

**DOI:** 10.1038/s41598-025-90452-y

**Published:** 2025-02-24

**Authors:** Gustaf Glavå, Birgitta Johansson

**Affiliations:** 1 Department of Psychology, University of Gothenburg, Box 500, Gothenburg, 40530 Sweden; 2 Institute of Neuroscience and Physiology, Department of Clinical Neuroscience, University of Gothenburg, Gothenburg, Sweden; 3 Department of Neurology, Sahlgrenska University Hospital, Gothenburg, Sweden

**Keywords:** Pathological mental fatigue, Acquired brain injury, Neurorehabilitation, MBSR, Qualitative evaluation, Brain injuries, Patient education

## Abstract

After acquired brain injury (ABI), some individuals suffer from long-term fatigue and emotional distress, which affects their work ability and daily life. Mindfulness-based stress reduction (MBSR) has shown promising results in quantitative studies as a complementary treatment for pathological mental fatigue (PMF) after ABI. This study aims to explore how people suffering from lasting PMF after ABI experience MBSR in relation to their PMF, with the intention of better meeting the rehabilitation needs of those affected. Seven individuals (mean age 42 years) who had suffered from long-lasting PMF after ABI took part in the study. None of them had resumed work due to their PMF, but all had recovered from neurological impairments. They were interviewed after completing an MBSR course. Thematic analysis of the participants’ perspectives suggested that the MBSR program provided them with coping techniques for living with PMF. They gained a deeper understanding of their condition becoming more self-compassionate, and the treatment provided them with a forum for meeting and sharing experiences with others with similar problems. The qualitative results strengthen the evidence for MBSR as a feasible psychoeducative complementary treatment for PMF after ABI.

## Introduction

Fatigue is a normal experience for all people, but after acquired brain injury (ABI)—such as stroke, traumatic brain injury (TBI), brain inflammation or brain tumors—residual mental fatigue is common^1–4^. Approximately 26–77% of individuals are estimated to suffer from fatigue after a stroke and 45–73% after a TBI; such fatigue is regarded as a primary symptom after ABI and can be distinguished from depression^1,5,6^. In addition, normal mental fatigue must be distinguished from pathological mental fatigue (PMF)^7^. The PMF syndrome includes rapid loss of perceived mental energy when the individual affected is exposed to stimuli—for example, reading a book or taking part in a conversation is difficult to manage over even a very short period—with a reduced ability to regain mental energy. The ability to concentrate is diminished, and the person becomes easily distracted. There is often clear variation in mental fatigue during the day, which is determined by the activity performed. Sleep and longer rest will not help but may provide some relief. Increased sensitivity to stress, as well as sensitivity to light and noise, is commonly reported, and emotional imbalance with tearfulness and irritability is common, as is impaired sleep, characterized by shorter and interrupted sleep or excessively prolonged sleep^8,9^. The ability to resume and manage work and ordinary daily activities is reduced, which is related to the inability to perform the same mental activity repeatedly^1,10,11^. Withdrawal due to PMF can be misunderstood as laziness or social reclusiveness, whereas it is a necessary means to avoid extreme exhaustion for individuals suffering from PMF^12^. Fatigue after ABI and neurological diseases can have substantial long-term negative effects on work ability, well-being, and quality of life^1,10,13^.

No efficient or recommended treatment exists for PMF following ABI, and only a few pharmacological treatment studies have been performed. A pharmacological feasibility study^14,15^ and one randomized control trial (RCT) with methylphenidate^16^ reported reduced fatigue. RCT studies with (-)-OSU6162 have shown mixed results for stroke and TBI^15,17–19^, while RCT studies with modafinil improved wakefulness but not fatigue^20,21^. In addition, improvements in fatigue after TBI have been shown in RCTs with blue light therapy^22,23^ and an adapted RCT using cognitive behavioral therapy^24,25^. Studies of the group-based mindfulness-based stress reduction program (MBSR) have produced promising results for people suffering from PMF after TBI, stroke and MS, with reduced mental fatigue and emotional distress as well as improved cognitive function^26–33^; two of these studies were RCTs^30,34^. However, recommended treatment options for fatigue are currently lacking within the health care system and are requested by those affected.

Mindfulness can contribute to well-being and could be a therapeutic option for those who have suffered an ABI^28,29,31,33,35,36^. The MBSR program has been used for 40 years^37^ and has clear guidelines regarding the course content as well as the quality of the certified MSBR teacher^37–39^. The primary intention of the MBSR program is to relieve suffering and increase wellbeing in the everyday lives of people with a wide range of medical and psychological conditions^37^. In addition, MBSR has shown promising results for individuals suffering from PMF after ABI and neurological illnesses^27,30^. The MBSR program is used in clinic and has been widely researched in numerous studies involving healthy and clinical populations^40–42^.

There is a growing body of quantitative studies on MBSR after ABI^43,44^. However, few studies have qualitatively explored the perspectives of participants suffering from fatigue after they attend MBSR programs. Reports of adapted mindfulness-based cognitive therapy (MBCT) have been documented in individuals with multiple sclerosis^45^ and chronic fatigue (MBCT)^46^. A study on mindfulness for ABI revealed that the mindfulness group benefited from learning mindfulness skills that were useful in everyday life, which increased their awareness and helped them cope with emotional and cognitive difficulties^33^. However, that study did not evaluate fatigue, and it did not involve a standard MBSR course. Therefore, further knowledge is needed concerning how people with PMF after ABI perceive the MBSR program since PMF could entail specific challenges in following the standard program. Adaptation of the program may thus be valuable. For example, people suffering from PMF easily experience mental exhaustion as a part of their condition, which could pose challenges when practicing mindfulness, as paying attention plays a central role in mindfulness^47^. Considering this potential challenge, it is intriguing that people suffering from PMF have been reported to benefit from MBSR^27,30^. Therefore, further knowledge is needed to gain a greater understanding of why MBSR can alleviate PMF. Perceptions of the benefits from those affected may have implications for understanding the underlying mechanisms by which MBSR may alleviate PMF. Such perceptions are unexplored among individuals suffering from PMF after ABI. Qualitative methods are suitable, as they enable the investigation of phenomena that remain scientifically unexplored^48^ and could serve to generate knowledge of the complexities involved in the therapeutic process and hence enhance the clinical usefulness of the program.

The aim of this study is to explore how people suffering from lasting PMF after ABI experience MBSR in relation to their PMF.

## Method

### Participants

Seven participants were recruited from an outpatient medical rehabilitation clinic for acquired brain injuries (ABIs). They were consecutively asked personally if they were interested in participating in the study after the formal rehabilitation period was finished. They had all previously received multiprofessional theme rehabilitation and were well aware of their PMF and the strategies to be used. The neuropsychologist and mindfulness teacher BJ informed participants about mindfulness and the MBSR program prior to the start of the study. All participants had the opportunity to practice meditation (40-min body scan) led by BJ for inpatients and outpatients at the neurorehabilitation clinic. None of the participants had previously taken part in an MBSR program.

Participants who met the inclusion criteria, i.e., experiencing persistent mental fatigue but not having a psychiatric disorder or severe cognitive or language impairments as assessed by neuropsychological tests, were invited to participate. Mental fatigue is the most common issue faced by patients at the neurorehabilitation clinic, who report similar symptoms of mental fatigue regardless of their diagnosis^49^.

The group included two men and five women, with a mean age of 42 years, who had completed high school or a university degree. All participants had suffered from PMF for more than one year after ABI (range 16–46 months), such as traumatic brain injury (TBI), stroke or benign brain tumor. They had recovered well cognitively and physically, and all were living independently, some with families and children.

None of the participants exhibited functional memory or verbal impairments, as determined by neuropsychological tests conducted during the rehabilitation period. Since these assessments were performed prior to the study and were not included in the ethical application, they are not reported here. None of them had resumed work due to their PMF. They reported a score on the Mental Fatigue Scale (MFS) well above the cutoff^49,50^ (Table 1). The MFS has been evaluated for people with ABI, with values above 10 indicating a significant problem with PMF. The MFS includes cognitive (concentration, memory, slowness of thinking), sensory (light and noise sensitivity) and emotional difficulties (tearfulness, irritability), as well as sleep problems. These symptoms are all commonly reported after neurological disorders^51^, and the symptoms of MFS have been shown to be closely related, exhibiting high internal consistency^49,50^.

**Table 1:** Background data of the participants, means, standard deviations (SDs), ranges and frequencies.

Participants (7)		Range
Age, years	41.9 ± 5.7	34–50
Gender	5 women/2 men	
Education	3 high school/4 university	
Employment status	all 0% work	
Diagnosis	3 TBI/3 stroke/1 benign brain tumor	
Time since injury, months	37 ± 12	16–46
Mental fatigue scale	20.2 ± 4.8	17–28.5

On the basis of clinical experience, severe memory, cognitive, or language impairments can hinder successful participation in an MBSR program and would have otherwise excluded participants from the study. However, it is common for individuals with PMF to report subjective difficulties with concentration and memory, which are closely associated with their PMF, whereas standard cognitive tests for memory and attention may fall within normal ranges^10^.

As an MBSR course spans eight weekly sessions, careful planning is required to ensure that the program is completed before longer holidays, such as summer or Christmas breaks. All the participants needed to begin the program simultaneously, and the seven participants in this study were those available and interested at the time of the study. We decided to proceed with this group of seven participants, all of whom shared a history of persistent PMF following ABI and had comparable background characteristics (Table 1).

### Procedure

All participants were formally (in writing) and informally (verbally) informed of the conditions of participation, and all gave their informed and written consent to participate. The study was approved by the Swedish Ethical Review Authority, protocol number 2021-000483. Among the ethical conditions were permission to withdraw from participation at any time during the study (no one did), secured treatment of the data, anonymity, and terms of data usage. To ensure anonymity, no names were written down at any stage of the study. Instead, coded names were used throughout the process. As the group was relatively small, the demographic information had to be presented in a nonindividual fashion. Presenting such information for each participant would have compromised anonymity. Owing to the COVID-19 pandemic, most interviews took place over Zoom, with the exception of one that was conducted in the neurorehabilitation center. The duration of the interviews ranged between 20–47 min and lasted for 33 min on average*.* The interviews were conducted within one week after the final session of the program. The interviews included questions concerning how the participants had experienced MBSR, whether and how they utilized information from MBSR, and other questions concerning MBSR in relation to PMF. Follow-up questions were asked to encourage participants to elaborate on their answers.

### MBSR program

The MBSR is a group-based program comprising eight weekly meetings, including a whole-day retreat. The meetings include discussions led by the teacher, with the purpose of exploring patterns of behavior, thinking and feeling and learning about alternative ways of coping with difficulties in life. MBSR is based on meditations such as body scans (designed to systematically, region by region, cultivate awareness of the body), sitting meditations for 20 and 45 min (awareness of the breath and systematic widening of the field of awareness to include awareness of the body, feelings of tone, mental states, and thoughts), breathing space and gentle Hatha yoga (with an emphasis on mindful awareness of the body). The participants are encouraged to take part in daily meditations of approximately 45 min and to complete weekly home assignments between the weekly sessions. The group setting offers the opportunity to share one’s experiences with others. The structure and standards of the MBSR curriculum were followed. The only adaptations were making sessions 2 h each due to the small number of participants and their PMF and shortening discussion and talks, with breaks involving short breathing meditations. The course was led by a certified MBSR teacher and clinical neuropsychologist who had met the participants previously in the clinic during their rehabilitation. The MBSR program was adapted to accommodate smaller class sizes, with a maximum of 10 participants in the group, in contrast to the larger groups typically permitted in standard MBSR sessions. This modification was made to reduce the burden on participants and better suit their needs. The standard curriculum was maintained.

### Measures

A semistructured interview was used to investigate the participants’ experiences with the MBSR intervention. The interview guide consisted of 18 questions. These included, for example, questions on how the participants had experienced the program in general and if and in what way the program had helped them deal with PMF. All interviews included spontaneous follow-up questions that encouraged the participants to elaborate on their responses.

### Data analysis

The aim of analysis was to explore how people suffering from lasting PMF after ABI experience MBSR in relation to their PMF. For this purpose, inductive semantic thematic analysis with a critical realistic approach was used^52^. That is, the participants’ responses were viewed as descriptors of their actual experiences while also considering that their descriptions are subject to contextual influences. ATLAS.ti (Version 23.0.1) was used for coding interviews, and multiple themes were coded in the extracts. The six phases of thematic analysis with revisions^52,53^ were conducted within this study as follows: In phase one, GG created verbatim transcripts of all interviews. These were read and reread by GG to familiarize himself with the material. In this phase, GG aimed to be open-minded with respect to the material, even though the focus of the analysis was to serve the aim of the study. In phase two, GG generated initial codes from all interviews. The codes were descriptions of excerpts from the interviews and thus represented patterns or content within the interviews that could be used to answer the research question. In total, 101 codes were generated. In phase three, GG merged codes from the initial coding. This process did not involve passively searching for emerging possibilities but rather actively generating clusters of codes. In this phase, GG refrained from finalizing the clusters; rather, he contemplated different versions. Upon completion of this phase, the 101 codes had been merged into 49 codes, of which 4 codes were based on at least 35 quotations each. In phase four, GG created a thematic structure on the basis of the phase three analysis to assess the fit of the themes in relation to both the code level of the data and the dataset as a whole. The main themes were created from three of the four clusters of codes with more than 35 quotes each that had been generated via code merging. This phase also involved reviewing whether the themes could be regarded as coherent and sound. The fifth phase related to the process of naming and defining the themes. In the sixth phase, GG selected relevant extracts from the transcripts to exemplify the themes and generate a descriptive account of how the themes corresponded with our aim. During this phase, both authors reviewed and discussed the themes, making final adjustments to theme names and descriptions to ensure that the results were coherent. Since the second author was the meditation teacher, their role was not to alter the analysis but rather to assess whether the descriptions were clear in relation to the quotes and theme names.

To provide transparency and explain the analytical process, we offer an example of the process from generating a code to later using it in the thematic structure. For example, the code “coping” was created to describe an excerpt from an interview, such as "I have used, like sitting meditation, a lot to manage, to get a little break from the pain experience." Subsequently, other excerpts could also be labeled as “coping” or assigned a different code that more accurately captured their content. In the case of the code "coping," it became part of the subtheme "intervention techniques," which was subsequently used to generate the overarching theme "MBSR as a coping technique for PMF."

The first author had prior training in thematic analysis through postgraduate-level courses and had previously published a paper utilizing thematic analysis in a peer-reviewed scientific journal. During the current analysis, the first author received feedback on coding procedures and thematic analysis from a senior lecturer in psychology with expertise in qualitative research methods. There was a concern that the authors’ preunderstanding of mindfulness treatments might influence the analysis. Specifically, the first author had participated in a nonclinical MBSR program, whereas the second author is a certified MBSR teacher with extensive clinical experience working with PMF patients in rehabilitation settings and conducting related research. To mitigate potential bias and separate the roles of MBSR teacher and researcher, the first author independently conducted and analyzed the interviews. To enhance neutrality, the first author refrained from reviewing the treatment material before conducting interviews or analyzing the data. This approach allowed for the collection and analysis of material without being directly influenced by the terminology or rationale associated with the treatment. After the first author had completed the initial analysis, both authors reviewed and discussed the findings together, reflecting specifically on the risk of bias. These discussions focused on ensuring that the analysis was grounded in the interviewees’ experiences rather than being shaped by the treatment material or the preexisting understandings of the researchers. In conducting and reporting this study, and specifically with respect to the concerns raised here, we adhered to the practices of reflexive thematic analysis^52^ to maintain rigor and transparency, ensuring that our research is communicated in a clear and comprehensible manner.

## Results

The aim of this study was to explore how participants suffering from lasting PMF after ABI experience the MBSR program in relation to their PMF. Analysis of the interviews generated three themes with seven subthemes that together provided a response to our research question. These themes and subthemes are presented in Table 2 along with example quotes for each subtheme. In our analysis, themes serve as descriptors of related subthemes^54^. The subthemes illustrate our analysis of the specific experiences that the participants described.

**Table 2:** Overview of themes, subthemes and example quotes.

Theme	Subtheme	Example quote
MBSR as a coping technique for PMF	Intervention techniques	“I have been able to use this if things become difficult in any way. I can walk away or can just stay in that environment but still shut myself in until I feel ready.” (IP2)
	Preventive techniques	“So, then I have tried to schedule these short breaks.” (IP1)
New perspectives	Understanding and accepting PMF through MBSR	“To understand your brain fatigue and be able to be more present in what one is actually doing, this being either together with others or by oneself, at home or away. Yes, to accept that it is so.” (IP2)
	Self-compassion	“Kinder, in the sense that I am not as harsh and judgmental and that is what we practiced here, this notion of having a kinder attitude towards oneself.” (IP3)
A forum to meet others with PMF	Exchanging experiences	“I think it is… it is kind of a two-sided effect to some extent … in terms of both the effect of the program and also a group effect where one, in some way shares experiences. I would never have achieved this effect if I had carried out the program myself.” (IP3)
	A chance to be normal	“Just this thing of having a place where one feels normal.” (IP6)
	Social challenges	“And then we sat in the same room, which I think everyone found quite stressful because then it became the very thing that is almost impossible to handle if you have PMF; that there are many conversations taking place in the same room.” (IP6)

### Theme 1: MBSR as a coping technique for PMF

In our analysis, this theme describes the participants’ perceptions of how the program had provided them with coping techniques for dealing with PMF. Thus, this theme addressed our aim by suggesting that, according to the participants’ experiences, the program had fulfilled their needs in terms of providing coping techniques for PMF. Our analysis revealed two types of techniques according to the experiences of the participants: (1) intervention techniques and (2) preventive techniques. The subthemes are described in greater detail below.

According to the analysis, the participants perceived that MBSR had provided them with techniques for intervention when they were about to exhaust themselves or when they felt the need for a break. For example, intervention could involve leaving a noisy social gathering and resting for a while before returning. It could also involve taking a short break with breathing-meditation to avoid exhaustion and to be able to last throughout the day. The example quote in Table 2 clearly illustrates the application of this technique.

In contrast to intervention techniques, preventive techniques involved trying to avoid exhaustion by anticipating the need for intervention. The quote in Table 2 illustrates a typical example of preventive techniques as the participant described scheduling breaks. The participants described how they scheduled both short breaks throughout the day and longer breaks, for example, in the afternoon between activities and family time. In our analysis, preventive techniques helped the participants take control of their condition and cope with PMF in their everyday lives. Interestingly, some participants described having adjusted their resting time from passive rest to MBSR exercises. They perceived that this switch had improved the quality of the rest, and they felt more in control of their rest.

### Theme 2: New perspectives

This theme describes the participants’ experiences of acquiring new perspectives through the MBSR program. This theme addressed our aim by conveying participants’ experiences of how the program provided an opportunity for participants to explore PMF through MBSR. Our analysis highlighted the following two aspects of new perspectives on the basis of the participants’ experiences: (1) an understanding and acceptance of PMF through MBSR and (2) self-compassion. The subthemes are presented in greater detail below.

According to the analysis, the MBSR program helped participants gain a better understanding of PMF and an acceptance of the condition with which they must learn to live. They gained new perspectives on PMF and its challenges through participating in the exercises and sharing their experiences. For example, participants described having become more attentive to certain needs and reaction patterns associated with PMF. As a result, some participants were thus able to accept the need for adjustments in their everyday lives to ensure compatibility with their PMF. Some participants described struggling to accept the need to adjust their lives. Instead, some spoke of acknowledging their needs, a word that we suggest is perceived as being more neutral than accepting PMF.

As illustrated in the example quote, another aspect of new perspectives concerned the notion of becoming more self-compassionate. The participants described that they had learned to be less judgmental and harsh, rather kinder, and more compassionate toward themselves after participating in the MBSR program.

### Theme 3: A forum to meet others with PMF

This theme describes the participants’ experiences with the MBSR program, which offered the opportunity to meet other people suffering from PMF. According to our analysis, the participants had an accumulated need to meet other people who shared their experiences with PMF. This need resulted from a common situation of being misunderstood by people not affected by PMF. In our analysis, MBSR as a forum for people with PMF included the following three aspects: (1) exchanging experiences, (2) having the chance to be normal and (3) social challenges. The subthemes are presented in greater detail below.

According to the analysis, participants experienced the MBSR program as a forum where they could exchange their experiences of living with PMF. As suggested in the example quote, being able to share experiences was a prominent aspect of how participants perceived the program and its positive effect on PMF. In fact, the program seemed to offer a first opportunity for several participants to meet others with PMF.

Moreover, sharing experiences and being among people with a similar condition was an opportunity to feel normal and to be understood within the group of participants with PMF, that is, a feeling of sharing and understanding among fellow participants in the course. This subtheme confirms that individuals with PMF can feel abnormal in their everyday lives because of their condition.

The opportunities afforded by practicing in a group also posed some social challenges. In our analysis, these challenges had to do with certain exercises in the program in which the participants had to talk to each other in pairs while they were all sitting in the same room. These types of exercises were experienced as challenging, as they involved having to sort through numerous sensory stimuli and being easily distracted. In addition, participants found traveling to and from the course by public transport challenging, and the same applied to having to socialize before and after the course with other participants. Nevertheless, some participants related that being in a group was not something that they had found challenging.

## Discussion

The aim of this study was to explore how people suffering from PMF after ABI experience the MBSR program in relation to their PMF, as this topic is unexplored. Previously, only quantitative studies of improvements in PMF have been reported for this group of individuals^26,27^. By interviewing individuals suffering from PMF who had participated in the MBSR program, we sought to answer the following question: What experiences do individuals with PMF following ABI have of MBSR in relation to their PMF? The results of this qualitative study demonstrated that the MBSR program provided participants with coping techniques for living with PMF; they had gained a deeper understanding of their condition and had become more self-compassionate. In addition, meeting others and the notion of gaining mutual understanding were greatly appreciated. We discuss these results in more detail below.

### MBSR as a coping technique for PMF

The results suggest that the MBSR program provided the participants with coping techniques for living with PMF. They were better able to adapt their activities to fit with their abilities and limitations. This did not entail avoiding activities but, rather, taking breaks, using short and long meditations and still taking part in social activities, but in new ways. Our results concerning MBSR as a coping technique for PMF are in line with previous studies on other clinical groups—although none were related to fatigue—that have described the use of meditative tools and practices learned from mindfulness-based interventions (MBIs, including MBSR and MBCT) as a means to address challenges related to medical conditions^33,45,55,56^. One interpretation of the results is that the coping techniques allowed participants to gain a sense of agency, emotional control, and self-efficacy in relation to their PMF. That is, the coping technique enabled them to feel more in charge of their PMF. According to our analysis, one set of coping techniques concerned the use of MBSR exercises to intervene in activities involved in daily life. This could entail taking a short break for breathing meditation or withdrawing from a noisy lunchroom. Arguably, these intervention techniques were also examples of how MBSR may have led to heightened self-efficacy among the participants. That is, the exercises allowed them to feel rooted in the present moment to gain mental control, thus serving as a protective mechanism for PMF.

PMF typically implies that the affected individual can utilize cognitive resources for short periods of time and then requires longer rest periods than normal to recover from exhaustion. This situation poses several challenges in relation to day-to-day activities^8^. By utilizing the intervention techniques, the participants created cognitive relief that allowed the brain to rest. In addition, the theme of preventive techniques described how a mindful approach was utilized as a general coping technique, providing ways to organize one’s life and to prevent exhaustion. In other words, the participants made use of mindfulness in a more preventive way rather than as an intervention technique in a specific situation. Such approaches could involve scheduling rest or planning the day in a way that is sustainable in relation to PMF. These experiences of the preventive use of mindfulness are in line with findings from previous studies on other clinical groups who experienced that mindfulness helped them prevent symptom expression and stress^55,57^. However, the alleviation of PMF by mindfulness has been previously reported only by quantitative studies^27,30^. We show here that different strategies as well as the use of mediations were helpful for the participants. Future endeavors could enhance our understanding of these findings by studying how the treatment is perceived in other groups and contexts. Such knowledge could provide insights into how to further adapt the treatment in relation to the experiences and needs of participants.

### New perspectives

The MBSR program gave the participants new perspectives on PMF, as they gained more knowledge and became more attentive to their needs and their reaction patterns. The theme of new perspectives illustrated how MBSR helped participants experience increased awareness of their medical condition and how this awareness, in turn, led to new perspectives on how to relate to and address these issues. Similar outcomes of group learning and new or altered insights into medical conditions have been shown in several qualitative studies on participant experiences of MBIs in which participants have described bonding, group support and shifting perspectives as outcomes of taking part in an MBI^55,57,58^. In addition to confirming these previous findings, this theme described several specifically relevant outcomes of MBSR in relation to PMF as experienced by the participants. The theme of new perspectives suggested that MBSR programs can increase awareness of behavior and cognitive patterns that are particularly negative when individuals are trying to cope with PMF, such as being mentally active too long, being in noisy environments and ruminating. In addition, the participants were able to find more kindness and self-compassion. This tendency can be viewed as interconnected with gaining an understanding and acceptance of PMF. That is, participants understand and accept how PMF limits and sets boundaries on everyday life and become more self-compassionate because of this greater awareness.

### A forum to meet others with PMF

According to the third theme, the MBSR program gave participants an opportunity to meet others with PMF and share their experiences. In addition, people with PMF commonly suffer from the invisibility of their condition, which can thus be misinterpreted as laziness or social reclusiveness whereas it is, in fact, a necessary means for coping with PMF. However, in the program, they shared these experiences and were able to support one another.

Sharing experiences with a group without having the need to explain shortcomings can be important and has been confirmed by previous studies, which have suggested that mutual understanding is a key aspect of building group solidarity and learning from others^46^. In addition, sharing experiences can improve learning and can serve to enhance mindfulness practices^59^. Practicing mindfulness in a group can help participants choose more appropriate and beneficial solutions and, when necessary, let go of control and of what one is holding on to.

### Benefits and difficulties

Researchers and practitioners need to be aware of the potential challenges of MBSR for individuals suffering from PMF, as our analysis has revealed. According to our analysis, reflecting in pairs offered both benefits and social challenges. These challenges stemmed from distractions from others in the room while trying to focus on discussion with the partner. Travel and socializing with other participants also proved challenging for some. However, not all participants found group activities to be problematic.

The participants had all been ill for a long time, had gained rehabilitation, were well aware of their PMF and had learned strategies for managing their daily activities. However, none of them had met others with PMF in a group activity during their rehabilitation. Meeting others, a sense of not feeling alone, and having an understanding—in the deeper sense of the word—was very much appreciated.

According to our results, MBSR is a feasible option for individuals suffering from PMF. They could follow the MBSR curriculum, and coming into contact with others with PMF, exchanging experiences and enhancing their insight and increasing their knowledge of their condition as well as learning how to cope with the problems it entails was helpful. However, it is necessary to adapt to individuals’ needs to fit with their PMF, as with the slower pace, less talking, more repetitions and the addition of more breathing meditations during the meetings, all of which are to be held in a safe and calm environment.

The MBSR curriculum was implemented with its standard long meditations and thematic structure, incorporating only minor adaptations such as a slower pace, shorter talks, and pauses when necessary, including brief breath meditations. Despite challenges such as reduced alertness and concentration difficulties, the participants expressed appreciation for the longer meditations and did not voice concerns about the duration of the sessions. The long meditations of 45 min have been used in our previous MBSR studies^26,27^, and our experience is that these long mediations can be maintained even if the participants suffer from PMF. However, some participants reported experiencing increased fatigue following the sessions, particularly after the full-day retreat. Longer meditations can provide individuals with time to calm their minds, reduce sensory impressions such as sounds and visual stimuli, and create an opportunity for mindful peace and rest. This practice—learning to distinguish between ordinary rest and "brain rest," where thoughts are quiet—along with repeatedly returning focus to one’s breath, may be beneficial for those suffering from PMF. Additionally, the MBSR program can help participants face challenges with reduced emotional reactivity. This is facilitated by the program’s inclusion of exercises and discussions that encourage individuals to explore alternative ways of responding to difficulties. However, for individuals with more severe or cognitive or language impairments or for those experiencing more pronounced PMF, the standard MBSR program may be too demanding. In these cases, a slightly shorter program with biweekly meetings may serve as a more appropriate alternative, as suggested for individuals with chronic fatigue^60^.

### Limitations

The current study is limited to the specific population it examined and the time span of eight weeks in which MBSR takes place. Other studies with follow-up interviews conducted later rather than alongside the MBSR program could result in variations in the results with respect to population variations, the frequency of continuous practice and other factors due to time elapsed. The interviews were conducted in conjunction with the program to avoid memory difficulties, which are common among individuals with ABI. This precautionary measure was reported in a previous interview study of individuals suffering from ABIs^33^. A relatively small number of participants were involved in the current study. This limitation implies that the analysis presented here could have been altered and made more divergent had more experiences from additional interviews been included. As highlighted in a study by van Ravesteijn et al.^61^, an attempt to extract deep and rich empirical data can be precarious when sample sizes are too small or too large. Arguably, the sample size in the current study was large enough to provide a breadth of analysis but small enough to allow a thorough analysis. Similar studies have also used small participant groups. For example, O'Dowd and Griffith^46^ conducted a qualitative study of nine participants with chronic fatigue.

Drawing on experiences from only one intervention group can be a limitation because it may lead to a narrow perspective, thus reducing the generalizability of the findings and potentially overlooking diverse experiences that could emerge from other groups. Additionally, the specific group context, including dynamics, relationships, and shared experiences, may unduly influence the results, making it difficult to separate the effects of the intervention from the unique characteristics of that particular group. These limitations should also be considered in relation to the current study and calls for further studies drawing on experiences from diverse groups. While we have taken measures to separate the roles of the investigator and MBSR teacher during the analysis process, we acknowledge that the dual role of the second author as both an investigator and the intervention teacher represents a potential limitation. To minimize social desirability bias in the analysis, the first author conducted all the interviews. The participants were informed that the second author, who was a neuropsychologist and MBSR teacher, would not listen to the interviews or read the transcripts in their entirety. Prior to the interviews, the participants were also informed that the transcripts would be anonymized and that the second author would not, at any stage, be able to identify which participant had provided specific responses.

## Conclusions

Owing to the long-term consequences of PMF that many people suffer from after ABI, there is a pressing need for treatment options. In this study, participants reported that MBSR provided coping techniques for PMF and a deeper understanding of their condition, and it was a valuable forum for meeting other people with PMF and sharing experiences. Our results, in combination with those of previous studies, suggest that the adapted MBSR program may be considered a valuable treatment option for individuals suffering from PMF after ABI. The qualitative results indicate that MBSR may be a feasible complementary treatment for PMF. To increase accessibility to the program for participants with more severe PMF, a shorter program with biweekly meetings may serve as a more appropriate alternative.

## Data Availability

The dataset generated and analyzed in the current study is not publicly available due to its sensitive nature. Anonymized excerpts from the interviews are available from the corresponding author on reasonable request.

## References

[1] Cantor, J. B. et al. Fatigue after traumatic brain injury and its impact on participation and quality of life. *J Head Trauma Rehabil* **23**(1), 41–51 (2008). 18219234 10.1097/01.HTR.0000308720.70288.af

[2] Staub, F. & Bogousslavsky, J. Fatigue after stroke: A major but neglected issue. *Cerebrovascular Diseases* **12**, 75–81 (2001). 11490100 10.1159/000047685

[3] Diaz-Arias, L.A., et al., *Fatigue in Survivors of Autoimmune Encephalitis.* Neurol Neuroimmunol Neuroinflamm, 2021. **November** (8): p. 6. 10.1212/NXI.0000000000001064 PMC8369511 34389660

[4] Asher, A., et al., *Fatigue among patients with brain tumors.* CNS Oncol. (2016) 5(2), 91–100, 2016. **5**(2): p. 91–100. 10.2217/cns-2015-0008 PMC6047436 26987038

[5] Kluger, B. M., Krupp, L. B. & Enoka, R. M. Fatigue and fatigability in neurologic illnesses. *Proposal for a unified taxonomy. Neurology* **80**(4), 409–416 (2013). 23339207 10.1212/WNL.0b013e31827f07be PMC3589241

[6] Western, E. et al. Fatigue After Aneurysmal Subarachnoid Hemorrhage: Clinical Characteristics and Associated Factors in Patients With Good outcome. *Front. Behav. Neurosci.* **15**, 633616 (2021). 34054441 10.3389/fnbeh.2021.633616 PMC8149596

[7] Skau, S., K. Sundberg, and H. Kuhn, *A Proposal for a Unifying Set of Definitions of Fatigue.* Frontiers in Psychology, 2021. **12**(739764). 10.3389/fpsyg.2021.739764 PMC8548736 34721213

[8] Johansson, B. & Rönnbäck, L. Long-Lasting Mental Fatigue After Traumatic Brain Injury – A Major Problem Most Often Neglected Diagnostic Criteria, Assessment, Relation to Emotional and Cognitive Problems, Cellular Background, and Aspects on Treatment. In *Traumatic Brain Injury* (ed. Sadaka, F.) 1–20 (INTECH, 2014).

[9] Rönnbäck, L. & Johansson, B. Long-Lasting Pathological Mental Fatigue After Brain Injury–A Dysfunction in Glutamate Neurotransmission?. *Front. Behav. Neurosci.* **15**, 791984. 10.3389/fnbeh.2021.791984 (2022). 35173592 10.3389/fnbeh.2021.791984 PMC8841553

[10] Johansson, B., Berglund, P. & Rönnbäck, L. Mental fatigue and impaired information processing after mild and moderate traumatic brain injury. *Brain Injury* **23**(13–14), 1027–1040 (2009). 19909051 10.3109/02699050903421099

[11] Eriksson, G. et al. Handling fatigue in everyday activities at five years after stroke: A long and demanding process. *SCANDINAVIAN JOURNAL OF OCCUPATIONAL THERAPY*10.1080/11038128.2022.2089230 (2022). 35758254 10.1080/11038128.2022.2089230

[12] Norrie, J. et al. Mild traumatic brain injury and fatigue: A prospective longitudinal study. *Brain Injury* **24**(13–14), 1528–1538 (2010). 21058899 10.3109/02699052.2010.531687

[13] Palm, S., L. Rönnbäck, and B. Johansson, *Long-term mental fatigue after traumatic brain injury and impact on capacity for workemployment status.* Journal of Rehabilitation Mededicine, 2017. **49**(228–233). 10.2340/16501977-2190 28150857

[14] Johansson, B. et al. Methylphenidate reduces mental fatigue and improves processing speed in persons suffered a traumatic brain injury. *Brain Injury* **29**(6), 758–765 (2015). 25794299 10.3109/02699052.2015.1004747

[15] Johansson, B. et al. Placebo-controlled cross-over study of the monoaminergic stabiliser (−)-OSU6162 in mental fatigue following stroke or traumatic brain injury. *Acta Neuropsychiatrica* **24**(5), 266–274 (2012). 25286991 10.1111/j.1601-5215.2012.00678.x

[16] Zhang, W.-T. & Wang, Y.-F. Efficacy of methylphenidate for the treatment of mental sequelae after traumatic brain injury. *Medicine* **95**(25), e6960 (2017). 10.1097/MD.0000000000006960 PMC5484184 28640076

[17] Berginström, N. et al. The Effects of (-)-OSU6162 on Chronic Fatigue in Patients With Traumatic Brain Injury: A Randomized Controlled Trial. *J Head Trauma Rehabil* **32**(2), E46–E54 (2017). 27120292 10.1097/HTR.0000000000000236

[18] Nilsson, M. K. L. et al. Effect of the monoaminergic stabiliser (−)-OSU6162 on mental fatigue following stroke or traumatic brain injury. *Acta Neuropsychiatrica* **32**(6), 303–312 (2020). 32418546 10.1017/neu.2020.22

[19] Western, E. et al. (−)-OSU6162 in the treatment of fatigue and other sequelae after aneurysmal subarachnoid hemorrhage: a double-blind, randomized, placebo-controlled study. *J Neurosurg* **136**(6), 1705–1715 (2021). 34715650 10.3171/2021.7.JNS211305

[20] Stankoff, B., et al., *Modafinil for fatigue in MS, a randomized placebo-controlled double-blind study.* 2005. **64**(7). 10.1212/01.WNL.0000158272.27070.6A 15824337

[21] Kaiser, P. R. et al. Modafinil ameliorates excessive daytime sleepiness after traumatic brain injury. *Neurology* **75**(20), 1780–1785 (2010). 21079179 10.1212/WNL.0b013e3181fd62a2

[22] Connolly, L.J., et al., *Home-based light therapy for fatigue following acquired brain injury: a pilot randomized controlled trial.* BMC Neurology, 2021. **21**(262). 10.1186/s12883-021-02292-8 PMC8256500 34225698

[23] Sinclair, K. L. et al. Randomized controlled trial of light therapy for fatigue following traumatic brain injury. *Neurorehabil Neural Repair* **28**(4), 303–313 (2014). 24213962 10.1177/1545968313508472

[24] Ponsford, J. et al. Factors Associated With Response to Adapted Cognitive Behavioral Therapy for Anxiety and Depression Following Traumatic Brain Injury. *J Head Trauma Rehabil* **35**(2), 117–126 (2020). 31365437 10.1097/HTR.0000000000000510

[25] Nguyen, S. et al. Cognitive Behavior Therapy to Treat Sleep Disturbance and Fatigue After Traumatic Brain Injury: A Pilot Randomized Controlled Trial. *Archives of Physical Medicine and Rehabilitation* **98**(8), 1508-1517.e2 (2017). 28400181 10.1016/j.apmr.2017.02.031

[26] Johansson, B. et al. Mindfulness-Based Stress Reduction (MBSR) Delivered Live on the Internet to Individuals Suffering from Mental Fatigue After an Acquired Brain Injury. *Mindfulness* **6**, 1356–1365 (2015).

[27] Johansson, B., Bjuhr, H. & Rönnbäck, L. Mindfulness based stress reduction improves long-term mental fatigue after stroke or traumatic brain injury. *Brain Injury* **26**(13–14), 1621–1628 (2012). 22794665 10.3109/02699052.2012.700082

[28] Azulay, J. et al. A pilot study examining the effect of mindfulness-based stress reduction on symptoms of chronic mild traumatic brain injury/postconcussive syndrome. *Journal of Head Trauma Rehabilitation* **28**(4), 323–331 (2013). 22688212 10.1097/HTR.0b013e318250ebda

[29] Bédard, M. et al. Pilot evaluation of a mindfulness-based intervention to improve quality of life among individuals who sustained traumatic brain injuries. *Disability and Rehabilitation* **25**(13), 722–731 (2003). 12791557 10.1080/0963828031000090489

[30] Grossman, P. et al. MS quality of life, depression, and fatigue improve after mindfulness training: a randomized trial. *Neurology* **75**(13), 1141–1149 (2010). 20876468 10.1212/WNL.0b013e3181f4d80d PMC3463050

[31] Acabchuk, R. L. et al. Therapeutic Effects of Meditation, Yoga, and Mindfulness-Based Interventions for Chronic Symptoms of Mild Traumatic Brain Injury: A Systematic Review and Meta-Analysis. *Appl Psychol Health Well Being* **13**(1), 34–62 (2021). 33136346 10.1111/aphw.12244

[32] Jani, B.D., et al., *Acceptability of mindfulness from the perspective of stroke survivors and caregivers: a qualitative study.* Pilot Feasibility Stud, 2018. **4**(57): p. 10.1186/s40814-018-0244-1. 10.1186/s40814-018-0244-1 PMC5827989 29497560

[33] Niraj, S., Wright, S. & Powell, T. A qualitative study exploring the experiences of mindfulness training in people with acquired brain injury. *Neuropsychol. Rehabil.* **30**(4), 731–752 (2020). 30230410 10.1080/09602011.2018.1515086

[34] Johansson, B. and L. Rönnbäck, *Mental Fatigue and Cognitive Impairment after an Almost Neurological Recovered Stroke.* International Scholarly Research Network Psychiatry, 2012. **2012**(Article ID 686425): p. 7 pages. 10.5402/2012/686425 PMC3658493 23738208

[35] Lawrence, M. et al. A systematic review of the benefits of mindfulness-based interventions following transient ischemic attack and stroke. *Int J Stroke* **8**(6), 465–474 (2013). 23879751 10.1111/ijs.12135

[36] Baldo, J. V. et al. Mindfulness-Based Stress Reduction Intervention in Chronic Stroke: a Randomized. *Controlled Pilot Study. Mindfulness* **12**, 2908–2919 (2021).

[37] Kabat-Zinn, J., *Full Full catastrophe living: How to cope with stress, pain and illness using mindfulness meditation.* 2001, London, 15th ed.: Piatkus Books.

[38] Crane, R. S. et al. Competence in Teaching Mindfulness-Based Courses: Concepts. *Development and Assessment. Mindfulness* **3**(1), 76–84 (2012). 23293683 10.1007/s12671-011-0073-2 PMC3395338

[39] Crane, S. R. Implementing mindfulness in the mainstream: making the path by walking it. *Mindfulness* **8**, 585–594 (2017). 28515799 10.1007/s12671-016-0632-7 PMC5408039

[40] Khoury, B. et al. Mindfulness-based stress reduction for healthy individuals: A meta-analysis. *Journal of Psychosomatic Research* **78**(6), 519–528 (2015). 25818837 10.1016/j.jpsychores.2015.03.009

[41] Kriakous, S. A. et al. The effectiveness of mindfulness-based stress reduction on the psychological functioning of healthcare professionals: A systematic review. *Mindfulness* **12**(1), 1–28 (2021). 32989406 10.1007/s12671-020-01500-9 PMC7511255

[42] Dobkin, P., Irving, J., & Amar, S, *For Whom May Participation in a Mindfulness Based Stress Reduction Program be Contraindicated?* Mindfulness, 2012. **3**(1): p. 44–50.

[43] Feng, Q., et al., *The Effects of Mindfulness-Based Interventions on Symptoms of Mild Traumatic Brain Injury: A Systematic Review.* International Journal of Mental Health Promotion 2024, 26(6), . , 2024. **26**(6): p. 417–428.

[44] Tao, S., et al., *Effectiveness of mindfulness-based stress reduction and mindfulness-based cognitive therapy on depression in poststroke patients-A systematic review and meta-analysis of randomized controlled trials.* J Psychosom Res, 2022. **Dec:163:111071**. 10.1016/j.jpsychores.2022.111071 36347179

[45] Bogosian, A. et al. *Potential treatment mechanisms in a mindfulness-based intervention for people with progressive multiple sclerosis* Brittish. *Journal of Health Psychology* **21**(4), 859–880 (2016). 10.1111/bjhp.12201 27238732

[46] O'Dowd, B. and G. Griffith, *"I Need to Start Listening to What my Body Is Telling Me.”: Does Mindfulness-Based Cognitive Therapy Help People with Chronic Fatigue Syndrome? .* Human Arenas, 2022. **5**(1): p. 5–25.

[47] Malinowski, P., *Neural mechanisms of attentional control in mindfulness meditation.* Frontiers in neuroscience, 2013. **7**(8). 10.3389/fnins.2013.00008 PMC3563089 23382709

[48] Creswell, J.W. and V.L. Plano Clark., *Designing and Conducting Mixed Methods Research*. Third ed. ed. 2017, Thousand Oaks, United States SAGE Publications Inc

[49] Johansson, B. & Rönnbäck, L. Evaluation of the Mental Fatigue Scale and its relation to Cognitive and Emotional Functioning after Traumatic Brain Injury or Stroke. *International Journal of Physical Medicine and Rehabilitation* **2**, 182 (2014).

[50] Johansson, B. et al. A self-assessment questionnaire for mental fatigue and related symptoms after neurological disorders and injuries. *Brain Injury* **24**(1), 2–12 (2010). 20001478 10.3109/02699050903452961

[51] Lindqvist, G. & Malmgren, H. Organic mental disorders as hypothetical pathogenetic processes. *Acta Psychiatrica Scandinavica* **88**(suppl 373), 5–17 (1993). 10.1111/j.1600-0447.1993.tb05611.x 8372702

[52] Braun, V. & Clarke, V. One size fits all? What counts as quality practice in (reflexive) thematic analysis?. *Qualitative Research in Psychology* **18**(3), 328–352 (2020).

[53] Braun, V. & Clarke, V. Reflecting on reflexive thematic analysis. *Qualitative Research in Sport, Exercise and Health* **11**(4), 589–597 (2019).

[54] Robinson, O. Relational analysis: An add-on technique for aiding data integration in qualitative research. *Qualitative Research in Psychology* **8**, 197–209 (2011).

[55] Malpass, A. et al. MBCT for Patients with Respiratory Conditions Who Experience Anxiety and Depression: A Qualitative Study. *Mindfulness* **6**(5), 1181–1191 (2015).

[56] Müller-Engelmann, M., et al., *Mindfulness-Based Stress Reduction (MBSR) as a Standalone Intervention for Posttraumatic Stress Disorder after Mixed Traumatic Events: A Mixed-Methods Feasibility Study.* Front Psychol, 2017. **8**(1407). 10.3389/fpsyg.2017.01407 PMC5591787 28928678

[57] Kvillemo, P. & Bränström, R. Experiences of a mindfulness-based stress-reduction intervention among patients with cancer. *Cancer Nursing* **34**(1), 24–31 (2011). 20555256 10.1097/NCC.0b013e3181e2d0df

[58] Visser, A. et al. A qualitative analysis of experiences of patients with metastatic breast cancer participating in a mindfulness-based intervention. *Palliative Medicine* **29**(2), 182–183 (2015). 25160693 10.1177/0269216314546206

[59] Cormack, D., Jones, F. W. & Maltby, M. A “Collective Effort to Make Yourself Feel Better”: The Group Process in Mindfulness-Based Interventions. *Qualitative Health Research* **28**(1), 3–15 (2018). 29017380 10.1177/1049732317733448

[60] McKechnie, F. *Mindfulness-Based Therapy for Managing Fatigue Supporting People with ME/CFS, Fibromyalgia and Long Covid* (Jessica Kingsley Publishers, 2023).

[61] van Ravesteijn, H. J. et al. Mindfulness-based cognitive therapy (MBCT) for patients with medically unexplained symptoms: process of change. *J Psychosom Res* **77**(1), 27–33 (2014). 24913338 10.1016/j.jpsychores.2014.04.010

